# Adaptation to large-magnitude treadmill-based perturbations: improvements in reactive balance response

**DOI:** 10.14814/phy2.12247

**Published:** 2015-02-03

**Authors:** Prakruti Patel, Tanvi Bhatt

**Affiliations:** Department of Physical Therapy, University of Illinois at Chicago1919 W, Taylor Street, 4th Floor., Chicago, Illinois, 60612

**Keywords:** Compensatory stepping, fall prevention, learning, slips

## Abstract

We aimed to examine the trial-to-trial changes in the reactive balance response to large magnitude slip-like treadmill perturbations in stance and whether the acquired adaptive changes could be appropriately scaled to a higher intensity perturbation. Seventeen young adults experienced 15 slips for training on level I intensity. Pre- and post-training slips were delivered at a higher intensity (20% > level I). Pre- and post-slip onset stability (at liftoff and touchdown of stepping limb) was measured as the shortest distance of the center of mass (COM) position (X_COM/BOS_) and velocity (Ẋ_COM/BOS_) relative to base of support (BOS) from a predicted threshold for backward loss of balance. The number of steps to recover balance, compensatory step length and peak trunk angle were recorded. The post-slip onset stability (at liftoff and touchdown) significantly increased across the trials with no change in preslip stability. Improvement in stability at touchdown positively correlated with an anterior shift in X_COM/BOS_ but not with Ẋ_COM/BOS_. Consequently, the number of steps required to recover balance declined. The adaptive change in X_COM/BOS_ resulted from an increase in compensatory step length and reduced trunk extension. Individuals also improved post-slip onset stability on a higher intensity perturbation post-training compared with the pre-training trial. The results support that the CNS adapts to fixed intensity slip-like perturbations primarily by improving the reactive stability via modulation in compensatory step length and trunk extension. Furthermore, based on prior experience from the training phase, the acquired adaptive response can be successfully calibrated to a higher intensity perturbation.

## Introduction

Falls are a common source of injury in the elderly resulting in rising healthcare cost. Approximately 40% of the falls causing serious injuries such as fractures occur due to slipping (Luukinen et al. 2000; Sterling et al. 2001). Owing to the growing population of older adults (≥65 years), an effective fall prevention intervention that can be implemented in clinical and community settings is of high priority. Several interventions such as gait and balance exercises (Liu-Ambrose et al. 2008; Clemson et al. 2012), Tai Chi (Logghe et al. 2009), general physical activity (Shigematsu et al. 2008) haven been implemented. Although these interventions have shown improvement in the lower extremity strength, physical function, and balance, whether they are effective in reducing the rate of real life falls is debatable (Kannus et al., 2005; Gillespie et al. 2012).

A novel but less widely used paradigm for preventing slip-related falls is perturbation training developed with the intention of providing task-specific adaptive training (Pai et al. 2003; Lockhart et al. 2005; Bhatt et al. 2006; Mansfield et al. 2010). Under this paradigm, individuals are exposed to several repetitions of forward directed perturbations during standing or walking simulating a real life condition such as slipping on the ice. Essentially, two methods have been used to deliver such perturbations - the ‘experimenter-controlled’ and ‘self-controlled’ perturbations. The self-controlled slips are often delivered using steel rollers or low friction movable platforms integrated in overground walkways (Marigold and Patla 2002; Bhatt et al. 2006) and low friction material surface such as a vinyl tile covered with oil (Brady et al. 2000). The intensity of perturbation can be controlled by altering walking speed or ankle dorsiflexion during landing (foot flat instead of heel strike to lower perturbation intensity). On the contrary, the experimenter-controlled perturbations are fixed intensity, delivered through computer controlled movable platforms (Mansfield et al. 2010) or treadmills (Grabiner et al. 2012; Yang et al. 2013).

The mechanism of adaptation from self-controlled perturbation is well-established in both young and elderly populations (Bhatt et al. 2006; Pai et al. 2010). These studies support that the central nervous system (CNS) uses the error information received from repeated perturbations and adapts by shifting from a feedback or reactive control to a feedforward or proactive control to produce protective responses (Pavol and Pai 2002; Pai et al. 2003; Bhatt and Pai 2005). After the first novel slip, given the knowledge of a possible slip over the upcoming trials, proactive adjustments are seen essentially in the slipping limb landing kinematics. The CNS adapts to maintain a more anterior center of mass (COM) state (position and velocity) during slip onset by reducing ankle dorsiflexion, increasing knee flexion, and reducing heel contact velocity influencing the ground reaction force resulting in a reduced perturbation intensity (Brady et al. 2000; Marigold and Patla 2002; Bhatt et al. 2006; Yang et al. 2009).

Relatively fewer studies report use of experimenter-controlled perturbations for training reactive balance responses to slip-like perturbations (Mansfield et al. 2010; Yang et al. 2013). As opposed to self-controlled perturbations, experimenter-controlled perturbations restrict an individual's ability to alter the perturbation intensity through proactive adaptations. Therefore, one might expect that recovering balance from experimenter-controlled perturbations demands higher reliance on the reactive adaptation via trunk control, and rapid compensatory stepping response to achieve stability. Previous research suggests that perturbation-based training in stance through motorized platform translations improved reactive balance by reducing the number of compensatory steps and mediolateral base of support (BOS) (Mansfield et al. 2010). Other researchers have also shown the potential of motorized treadmill-based perturbation training for reducing falls among younger and older adults (Shimada et al. 2004; Grabiner et al. 2012; Yang et al. 2013) and in individuals with Parkinson's disease (Protas et al. 2005). Although previous studies provide some evidence supporting use of experimenter-controlled perturbations for improvement in the reactive balance control and fall outcomes, the exact mechanism of adaption to such perturbations (change over trials) has not been examined and is not completely understood yet.

It is postulated that the CNS possesses an internal representation of body's COM in relation with the BOS based on prior experience (Pai and Iqbal 1999; Pai 2003). Thus, when exposed to an external perturbation, the CNS would compare the actual and desired COM state to execute a reactive motor response through a coordinated trunk and lower limb movement to achieve a stable position. After the initial exposure, with subsequent perturbations, the CNS would update this internal representation and learn to scale the magnitude of response based on the error information received from former trials (Horak and Diener 1994), resulting in an improved response on subsequent trials. Such exposure if provided for adequate amount (sufficient number of repetitions) could induce motor learning such that the acquired responses could be retained and transferred to similar contexts (Bhatt and Pai 2009; Parijat and Lockhart 2012; Wang et al. 2011). However, it remains to be determined if such postulation holds true for large scale experimenter-controlled perturbations. As the reactive responses are preferred and main line of defense for balance control against sudden large magnitude perturbations, training these responses is crucial for preventing an actual fall.

Thus, the objective of this study was to examine the adaptive changes in the reactive balance control associated with exposure to repeated fixed intensity treadmill slip-like perturbations in stance and to test if the acquired adaptive changes could be appropriately scaled to a higher intensity untrained perturbation (20% more than training intensity, Level II). We hypothesized that with perturbation training (on level I) subjects would show an initial adaptive phase (from trial 1 to trial 10), followed by a plateau phase (from trial 10 to trial 15) of improvement in the reactive (post-slip onset) stability resulting in decreased number of compensatory steps required to recover balance. The enhanced reactive stability would be achieved by significant increase in compensatory step length and reduction in trunk extension (from trial 1 to trial 15). We also hypothesized that the subjects would demonstrate successful scaling of the acquired adaptive changes to a higher intensity indicated by significantly improved performance (reactive stability) on the post-training compared with the pre-training level II perturbation.

## Methods

### Participants

The study was approved by the institutional review board of the University of Illinois at Chicago. Seventeen healthy young adults (M* *=* *23.47 years*,* SD *= *2.93; three males and 14 females) participated in the study. All the subjects with a self-reported history of any musculoskeletal injuries, cardiopulmonary, or neurological disorders were excluded.

### Reactive balance testing and training

**Figure 1:**
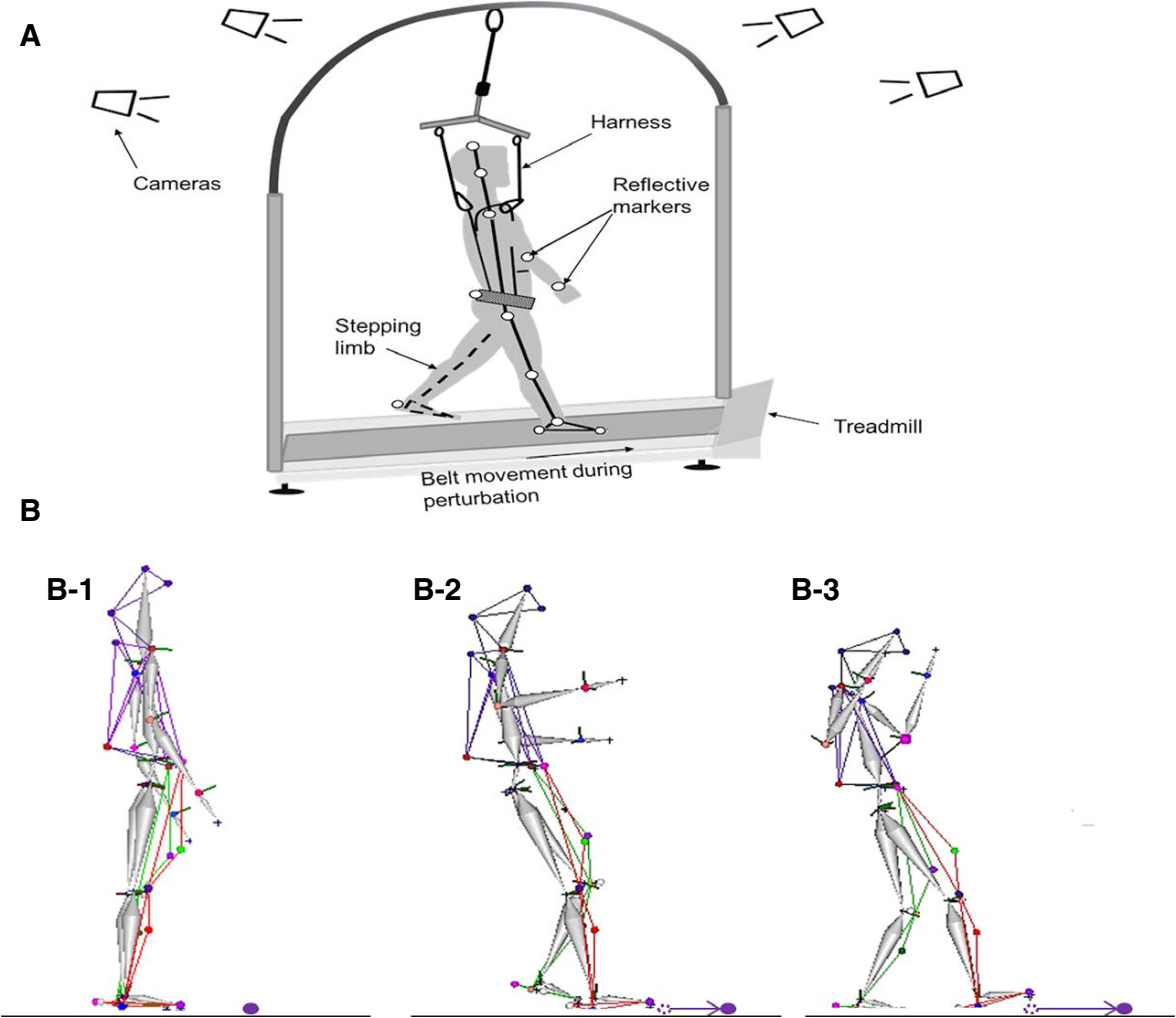
(A) Schematic representation of the experimental set up and (B) compensatory stepping response to slip-like perturbation of a representative subject during the time events of B1) slip onset, B2) liftoff of the stepping limb (LO), and B3) touchdown (TD) of the stepping limb. During LO and TD, the initial position of the belt marker is indicated by the empty circle and the arrow represents direction of the belt displacement.

**Figure 2:**
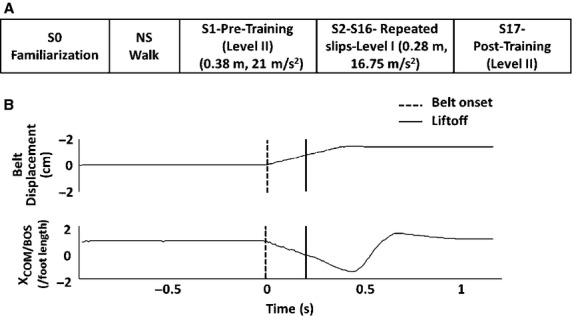
Figure showing (A) Experimental protocol – trials S2 to S16 constituted the training session, and trials S1 and S17 were the pre- and post-training slips delivered at a higher intensity compared to the training session. The S0 and S1 trials were separated by a regular walking trial (NS). (B) A typical trajectory of belt displacement during perturbation (top) and center of mass displacement relative to base of support normalized to the foot length (X_COM/BOS_/foot length, bottom).The vertical lines represent the events of belt displacement onset (dotted) and liftoff of the stepping limb (solid).

Forward perturbations were induced in standing position through a microprocessor controlled ActiveStep Treadmill, (Simbex, Lebenon, NH). Individuals stood at the center of the treadmill assuming a comfortable stance position with feet placed approximately shoulder width apart. A safety harness prevented the subjects’ knees from touching the treadmill belt in case of a fall (Fig.1A and B). They were instructed that at any given time instance; the treadmill belt would suddenly translate forward without their prior knowledge. The subjects were asked to respond in a natural way to recover balance and prevent themselves from falling. After receiving one familiarization trial, all the subjects walked on the treadmill at comfortable walking speed for one min. The walking trial served as a washout trial in order to record subject's natural response on the pre-training perturbation trial. The first pre-training slip (forward perturbation) in standing position was then delivered with a velocity of 0.86 m/s, for 0.38 m, and acceleration of 21 m/s^2^ (Level II intensity). The pre-training slip was followed by a block of 15 repeated slips at a lower intensity with a displacement of 0.19 m at 0.67 m/s, and acceleration of 16.75 m/s^2^ (Level I intensity, 20% less than level II). A 20% increase in perturbation intensity was chosen based on the previous studies reporting intralimb transfer of adaptive changes in response to upper extremity perturbations (Morton et al. 2001) and training for locomotor perturbations (Grabiner et al. 2012). An increment of intensity by 10% might not be sufficiently challenging to truly test the transfer of adaption. Following 15 slips at a lower intensity, without subjects’ prior knowledge, a post-training slip at level II intensity was delivered (Fig.2A). At the beginning of the session, subjects were informed that they would be exposed to repeated 20–25 slips, however; they were unaware of the intensity of all the slips delivered.

### Data collection

Full body kinematics was measured using an eight-camera motion capture system recording at 120 Hz (Motion Analysis, Santa Rosa, CA). Helen Hayes marker set with 29 reflective markers for head, trunk, upper extremity, and lower extremity were used to record full body kinematics and compute the COM. One marker was placed on the treadmill belt which served as an identifier for the perturbation onset. A sample representative trace of treadmill belt displacement and COM position relative to the BOS during perturbation is shown in Fig.2B. All the trials (*n* = 3) with missing markers were excluded from the analysis.

### Data analysis

Stability: The COM position in the anteroposterior direction was expressed relative to the most posterior heel position (X_COM/BOS_) and was normalized to the foot length. The COM velocity was computed by the first order differentiation of the COM position and was normalized to a dimensionless fraction of √*g*x*h*, *g* being the acceleration due to gravity and *h* is the body height (McMahon 1984). The COM velocity was expressed relative to the velocity of the most posterior heel marker of the BOS (Ẋ_COM/BOS_) (Bhatt et al. 2006)_._ Thus, a more negative X_COM/BOS_ and Ẋ_COM/BOS_ indicate that the COM is posterior to the BOS with a slower velocity contributing toward instability in the backward direction. The COM state stability was computed by comparing the instantaneous COM position (X_COM/BOS_) and velocity (Ẋ_COM/BOS_) to a previously published computational boundary for the threshold for backward loss of balance (Pai and Iqbal 1999). The COM state stability values of <1 indicate instability in the backward direction. Thus, a more negative value is associated with greater instability in the backward direction. The X_COM/BOS_, Ẋ_COM/BOS_, and stability were recorded 100 ms prior to the onset of the perturbation (pre-slip), and after the perturbation onset at the liftoff (LO) and touchdown (TD) of the stepping limb (post-slip) (Bhatt et al. 2006). Furthermore, the degree of loss of balance was assessed by the number of compensatory steps taken to recover balance. The individuals were said to have demonstrated two or more steps when the perturbation evoked more than one backward step to recover balance, with the second step landing posterior to the first step.

Other spatial variables: To examine the mechanism of improvement in stability, the compensatory step length and trunk angle were recorded. The compensatory step length was measured as the distance between the stepping limb heel position at the perturbation onset and TD of the compensatory step. It was then normalized to the individual's body height. The peak trunk angle relative to the vertical orientation in the sagittal plane was calculated between the events of perturbation onset and TD of the compensatory step such that zero degree represents a vertical orientation. More positive values indicate trunk flexion whereas negative values indicate trunk extension. The events of compensatory step LO and TD were determined from heel marker coordinates in the anteroposterior direction (Oates et al. 2010).

### Statistics

A Friedman's test was performed to analyze trial-to-trial changes in the number of steps taken to recover balance. Post hoc Wilcoxon signed rank test was used to analyze differences in the number of steps between trials 1, 10, and 15. To assess the effect of the first novel exposure to the perturbation on the subsequent pre-training trial, a paired *t-*was performed on stability between the familiarization and the pre-training trial. A 3 × 3 repeated measures ANOVA was performed to examine the trial-to-trial change in stability across trials 1, 10, and 15 as a function of the events: preslip onset and post-slip onset at LO, and TD of the stepping limb. The significant main effects of the trials and events and any significant interactions (trial × event) were resolved using paired *t-*tests after controlling for multiple comparisons (Bonferroni's correction). A regression analysis was performed to examine the relationship across trials between stability, and the X_COM/BOS_ and its Ẋ_COM/BOS,_ at post-slip onset TD of the stepping limb.

To determine the mechanism of adaption in stability, a linear regression was performed between stability at TD, and compensatory step length and peak trunk angle. To evaluate the scaling of the adaptive changes of training on a lower intensity to a higher intensity slip, paired *t-*tests were performed between the pre-training and post-training stability. The difference in number of steps pre- and post-training was analyzed using the Wilcoxon signed rank test.

## Results

### Adaptive changes in reactive stability

All the subjects demonstrated a backward compensatory stepping response upon sudden forward perturbation. The number of steps implemented to recover balance declined across the trials which demonstrated a significant exponential fit (*r*^2^* *= 0.88, *P *<* *0.05). A significant main effect of trials was observed *χ*^2^ = 64.14, *P *<* *0.05. Post hoc comparisons using the Wilcoxon signed-rank test indicated that the number of steps reduced from trial 1 to trial 10 (*P *<* *0.05), and from trial 1 to trial 15 (*P *<* *0.05), with no further decline from trial 10 to trial 15. Such a decline in number of steps was accompanied by an improvement in the post-slip stability at LO and TD.

**Figure 3:**
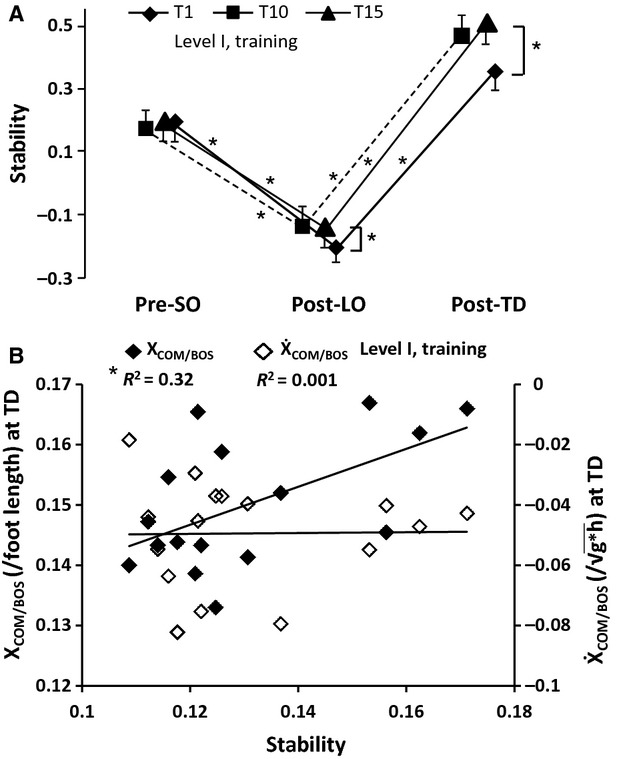
Figure showing (A) adaptation in proactive, preslip onset (Pre-SO) and reactive, post-slip onset stability at liftoff (Post-LO) and touchdown (Post-TD) of the stepping limb for trial 1 (T1), trial 10 (T10), and trial 15 (T15) and (B) relationship between the stability at touchdown (TD) and the center of mass position and velocity relative to the base of support (X_COM/BOS_ and Ẋ_COM/BOS_ respectively). The stability was defined as the shortest distance of the instantaneous COM state (position and velocity) from a theoretically predicted threshold for backward loss of balance. Stability values <1 indicate instability in the backward direction. Significant differences are indicated by *P <* *0.05.

Overall, there was a significant main effect of trial on stability across the events *F*_2,52_ = 3.81, *P *<* *0.05 (see Fig.3A). Although there was no significant difference in preslip stability across the trials (*P *>* *0.05), there was a significant improvement in post-slip stability at LO and TD of the stepping limb from trial 1 to trial 10 (*P *<* *0.05 for both comparisons) with no further improvement from trial 10 to 15 at both LO and TD (*P *<* *0.05). The stability during all the three events also differed among trials 1, 10, and 15 [*F*_2,82_ = 192.10, *P *<* *0.05]. For all the three trials, the stability significantly declined from preslip to LO of the stepping limb followed by an improvement from LO to TD of the stepping limb (*P *<* *0.05 for all comparisons). For trial 1, the stability at TD was lower compared with the preslip stability (*P *<* *0.05), however; there was no significant difference in stability between the two events for trials 10 and 15 (*P *>* *0.05) Furthermore, a linear regression analyses demonstrated a significant positive correlation between the stability and X_COM/BOS_ at TD (*r*^2^ = 0.32, *P *<* *0.05) such that an increase in stability was associated with an anterior shift in X_COM/BOS_ at TD, however, there was no correlation between the stability and 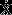
_COM/BOS_ at TD (*r*^2^ = 0.001, *P *>* *0.05) (see Fig.3B).

**Figure 4:**
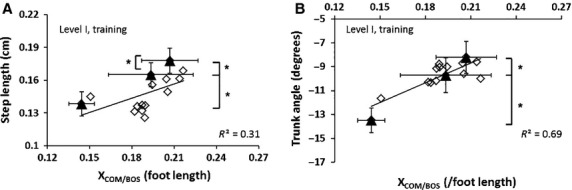
Scatter plot showing the relationship between stability at touchdown (X_COM/BOS_ at TD) with (A) compensatory step length and (B) trunk angle. Significant associations between the variables are denoted by *P <* *0.05.

The improvement in X_COM/BOS_ at TD was associated with a corresponding change in the compensatory step length and peak trunk angle. The X_COM/BOS_ at TD positively correlated with compensatory step length (*r*^2^* *= 0.31, *P *<* *0.05, Fig.4A). The subjects increased compensatory step length with repeated exposure to perturbations *F*_1,16_ = 6.00, *P *<* *0.05, showing a significantly larger step length from trial 1 to trial 10 and from trial 10 to trial 15 (*P *<* *0.05 for both comparisons). The anterior shift in X_COM/BOS_ at touchdown also correlated with reduced peak trunk extension across the trials (*r*^2^ = 0.69, *P *<* *0.05, Fig.4B). A significant main effect of trials was observed for peak trunk extension *F*_1,16_ = 5.27, *P *<* *0.05. In particular, the peak trunk extension reduced from trial 1 to trial 10 and from trial 10 to trial 15 (*P *<* *0.05 for both comparisons).

### Scaling of adaptive changes to a higher intensity perturbation

**Figure 5:**
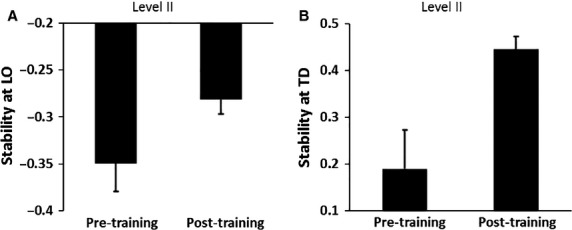
Figure demonstrating mean differences in post-slip onset stability between the pre-training (Pre) and post-training trials on a higher intensity perturbation. *P <* *0.05 represents the differences in stability at (A) liftoff (LO) and (B) touchdown (TD).

In comparison with the familiarization trial, there was a trend toward increase in stability at stepping limb TD, however, this did not reach the level of significance *t*_14_ = −1.94, *P *>* *0.05 (M* *=* *−0.02, SD* *= 0.25 for familiarization trial, M* *=* *−0.02, SD* *= 0.09 for pre-training trial). Compared with the pre-training trial (on Level II intensity), on the post-training trial at the same intensity, the subjects reduced the number of steps taken to recover balance *z* = −2.96, *P *<* *0.05 (M* *=* *2.13, SD = 0.56 for pre-training and M* *=* *1.07, SD = 0.26 for the post-training trial). Furthermore, the stability at both LO and TD of the stepping limb significantly increased on the post-training compared with the pre-training trial, *t*_14_ = −3.67 and *t*_14_ = −2.40, *P *<* *0.05 respectively (Fig5). The im-provement in postural stability at TD was accompanied by increase in compensatory step length *t*_17_ =−2.56, *P *<* *0.05 (Fig5c) however, there was no difference in trunk extension *t*_17_ = −0.92, *P *>* *0.05.

## Discussion

We investigated the adaptive changes following exposure to repeated slip-like treadmill perturbations. As hypothesized, the subjects improved their reactive (post-slip onset) stability with repeated exposure to perturbations. Specifically, there was a significant improvement from trial 1 to trial 10 that was maintained from trial 10 to trial 15. The fact that there was no change in stability prior to perturbation onset reveals that the central nervous system (CNS) adapted predominantly by improving the reactive (post-slip) control of stability.

It is proposed the COM state (position and velocity relative to the BOS) contributes toward the stability control (Pai and Iqbal 1999; Pavol and Pai 2002; Pai et al. 2010; Parijat and Lockhart, 2012). In case of sudden slip-like perturbation, when the perturbation intensity can be controlled by the subjects, it often results in emergence of proactive (preslip onset) adaptations for reducing the perturbation intensity which subsequently influences the reactive (post-slip onset) stability and the overall slip outcome (Marigold and Patla 2002; Bhatt et al. 2006; Oates et al. 2010). We, however, observed that the improvements in stability were largely accounted for by reactive changes in stability with no significant change in proactive stability. Such an adaptive response was induced by an anterior shift in the COM position relative to the BOS with little change in COM velocity. It is plausible that the inability to modulate the perturbation intensity could have made it challenging to alter the COM velocity in order to control stability before perturbation onset. A change in the COM position could have been more easily achieved via a modulation of compensatory step kinematics and trunk angle. Also, given the standardized starting position in the stance condition for all subjects, it is possible that anticipatory changes were minimized.

Overall, the reactive adaptation to slip-like perturbations could be accounted for by two main mechanisms. Specifically, trial-to-trial increase in compensatory step length and reduced peak trunk extension with repeated exposure to perturbations contributed towards the anterior shift in X_COM/BOS._ While reducing the trunk extension controlled the excessive COM excursion in posterior direction, increasing the extent of BOS provided a greater area for COM excursion without experiencing loss of stability. (Hsiao and Robinovitch 1998; McIlroy and Maki 1999). Also, the increase in compensatory step length could have contributed towards enhanced stability by providing a greater stabilizing moment through the contact force to decelerate the COM excursion (Maki and McIlroy 1997).

As a result of improved stability at TD, the number of steps exponentially reduced across all the trials. Implementing multiple steps to recover balance has been linked with poor balance control reflecting inability to arrest the COM displacement leading to a higher risk of falling (McIlroy and Maki 1996). Thus, a decline in number of steps required to recover balance shows the CNS's ability to employ a feedback mechanism to update the error information from preceding trials in order to improve stability over the subsequent trials (Horak and Nashner 1986; McIlroy and Maki 1999; Pai 2003).

It is proposed that the adaptive response to perturbation is largely influenced by the ‘central set’ developed from one exposure to perturbation (Timmann and Horak 1997). Thus, during initial exposure to a sudden slip-like postural disturbance, the CNS would have employed a feedback response to execute a compensatory step to maintain stability (Diener et al. 1988; Winstein et al. 1991). However, with repeated exposure to the slip-like perturbations, the initial feedback response might be recalibrated to refine and select a triggered response on the subsequent trials. Triggered responses are fast prestructured coordinated responses which can be triggered into action in response to similar stimulus by bypassing some stages of information processing (Schimidt and Lee 1999). It involves executing a response by refining or recalibrating a previously formed motor memory without the need of developing a new motor memory. A memory for reactive balance response could already be existing due to the perturbations experienced over one's life span which was further primed by perturbation training. Thus, it can be suggested that as the individuals improved their stability with repeated exposure to perturbations, the CNS would have triggered a previously formed response using a new trajectory each time to correct the error from previous trial to attain an optimal level of stability. This was evident through improvement in stability by increase in compensatory step length and reduction in peak trunk extension until a predetermined stability state was achieved (Winstein et al. 2000).

In line with our second hypothesis, the subjects showed a successful scaling of the adaptive skills acquired on a lower intensity perturbation (level I) to a higher intensity perturbation (level II). The stability at both LO and TD post-training improved compared with pre-training trial on a higher intensity along with reduction in the number of steps required to recover balance. Empirical evidence suggests that the contextual information is essential for selection of an appropriate motor response based on the sensorimotor feedback received (Wolpert and Kawato 1998). Past studies on locomotor perturbations have demonstrated that recovering from perturbations delivered in quick succession leads to the development of a motor memory specific to slip-like perturbations, nevertheless can be applied to situations altering certain features of the perturbation (Pai et al. 2003; Bhatt and Pai 2008; Grabiner et al. 2012). In our study, the fact that the higher intensity perturbation was delivered with the same treadmill, subjects were familiar with the contextual information. Based on prior experience, the subjects were thus able perceive the change in the sensorimotor information i.e. the perturbation intensity and execute the necessary motor response acquired during the adaptation phase to achieve a better reactive stability on a higher intensity perturbation than their first exposure. Calibration of the adaptive changes acquired on a lower intensity to a higher intensity perturbation thus indicates that the CNS selects a favorable motor response from the newly formed motor memory based on the perceived contextual information(Imamizu et al. 2007).

Researchers have shown that cerebellum through connections to the brainstem is involved in control and adaptation of balance and locomotor tasks (Timmann and Horak 1997; Morton and Bastian 2004) however, postural responses from large-scale perturbations are also cortically modulated. This evidence comes from behavioral (dual-task paradigms) and neurophysiological (imaging and cortical activations) studies (Maki et al. 2001; Brauer et al. 2002; Solopova et al. 2003). For example, recent line of evidence from animal and human research has shown direct activation of motor cortex for initiating and executing corrective (reactive) responses to external perturbations (Adkin et al. 2006; Beloozerova et al. 2003, 2005; Deliagina et al., 2007). Hence, although the response to a perturbation involves both short and long-loop reflexes within the spinal cord and brainstem levels, it can be proposed that initial learning of the reactive responses might be regulated by cerebellar connections to the cortex (Graydon et al. 2005).

Training reactive responses can have significant clinical implications for fall prevention in older adults and individuals with neurological impairment – populations that are at a higher fall risk. While several studies have shown improved laboratory fall outcomes post overground perturbation training, one of the pressing concerns limiting the use of this technique is lack of direct translation to community and clinical settings given the need for sophisticated equipment and large space. On the other hand, motorized treadmills being compact, cost-effective, and portable can have a widespread use in these settings.

With regards to current findings, it is proposed that through repeated exposure to such perturbations, CNS develops a specific motor memory to improve reactive stability predominantly through a feedback mechanism to maintain more forward COM position when re-establishing the BOS by a compensatory stepping response. Reactive stability is further enhanced through online recalibration of the newly formed motor memory for parameterization of step length and trunk extension in order to maintain more forward COM position. These results also support the CNS's ability to alter the learnt motor skills to the magnitude perturbation by integrating the perceived sensorimotor information with prior experience.

## Conflict of Interest

None declared.
